# Retrospective Cohort Study Comparing Different Hysterectomy Approaches for the Treatment of Endometrial Cancer

**DOI:** 10.3390/cancers18121977

**Published:** 2026-06-18

**Authors:** Anisha Dubey, Maria Huichochea Munoz, Julia Kobylianski, Mahshid Hosseini, Melody Wyslobicky, Jessica Pudwell, Anita Agrawal

**Affiliations:** Division of Gynecologic Oncology, Department of Obstetrics and Gynecology, Queen’s University, Kingston, ON K7L 3N6, Canada

**Keywords:** endometrial cancer, oncology, minimally invasive surgery, surgical approach, laparoscopy

## Abstract

**Background**: Endometrial cancer is the most common gynecologic malignancy in North America, with over 8000 new diagnoses in 2022 alone. Surgical management is the mainstay of treatment, with a shift towards the use of minimally invasive surgery, including laparoscopic and robotic techniques. Therefore, our study aims to compare the surgical and oncologic outcomes of hysterectomy approaches for patients with endometrial cancer. **Methods**: We have used a retrospective design to review all the endometrial cancer cases from Kingston Health Sciences Centre between January 2017 and November 2022. Variables were collected and categorized. Surgical outcomes were compared based on the route of surgery. Kaplan–Meier and Cox proportional hazard models were used to compare recurrence-free and overall survival. **Results**: A total of 341 cases were included in this review. One-hundred-fifty-two (44.6%) of these cases were minimally invasive surgeries (total laparoscopic, laparoscopic-assisted vaginal and robotic-assisted hysterectomies) and 189 (55.4%) were open hysterectomies. The early complication rates (*p* < 0.001) and length of stay (*p* < 0.001) were lower in the laparoscopic group. Despite the abdominal group including more advanced cases, there were no differences in recurrence-free and overall survival between the groups (*p* = 0.39; *p* = 0.85). **Conclusions**: Minimally invasive hysterectomy approaches result in improved surgical outcomes, while oncologic outcomes remain similar across techniques.

## Simple Summary

There are multiple ways to complete a hysterectomy, including vaginal, laparoscopic, robotic and open approaches. Our study aims to determine which surgical approach for patients with endometrial cancer best minimizes the risk of recurrent disease as well as surgical morbidity. We reviewed over 300 hysterectomy cases completed at Kingston Health Sciences Centre in Ontario, Canada, and categorized them based on the type of surgery performed. Patients who underwent minimally invasive surgeries were grouped together and compared to those who had an open hysterectomy. Survival and recurrence data was collected and analyzed. Our results suggest that, regardless of the surgical approach, the surgical and oncological outcomes remain the same.

## 1. Introduction

Endometrial cancer is the most common gynecologic malignancy in North America, with an increase in incidence of 50.0/100,000 cases between 1990 and 2019 [1]. This is thought to be due to the increased prevalence of obesity-related risk factors as well as the declining rate of hysterectomy for benign causes [2]. Initial management for endometrial cancer includes surgical resection and staging to estimate patient prognosis and assess the need for adjuvant therapy. The standard surgical treatment includes a total hysterectomy and bilateral salpingo-oophorectomy [3]. Additional surgical procedures, such as sentinel lymph node dissection in early-stage endometrial cancer, vary across countries [4,5,6]. In Canada, sentinel node dissection has been adopted by most centres and is part of routine surgical treatment for grades 2 and 3 endometrial adenocarcinoma as well as higher-grade histologies. More advanced surgical procedures, such as para-aortic lymph node dissection or omentectomy, can be performed depending on the suspected histology of the tumour [5].

Traditionally, a hysterectomy was performed through a laparotomy or open approach, known as a total abdominal hysterectomy (TAH). Minimally invasive approaches, including total laparoscopic hysterectomies (TLHs) and robotic-assisted hysterectomies (RAHs), are alternatives to a TAH and result in shorter hospitalizations and faster recoveries [7,8,9,10,11]. A randomized controlled trial conducted by Mourits et al., in 2010 reported that patients who underwent a TLH compared to a TAH had significantly less pain medication use, shorter hospital stay, less intraoperative blood loss and faster recovery [10].

Similarly, minimally invasive surgical techniques have been associated with fewer postoperative complications in multiple randomized trials and systematic reviews [8,11,12,13]. For instance, Park et al., published a systematic review demonstrating equivalent rates of intraoperative complications across the three surgical techniques, with lower likelihood of conversion to laparotomy with RAH compared to TLH [12].

With regard to oncological outcomes, a 2023 systematic review published by Fu et al., showed equivalent rates of overall survival (OS) and disease-free survival between patients who underwent RAH and TLH; however, OS and disease-free survival were higher in the RAH and TLH groups compared to the TAH group. Specifically, for early-stage endometrial cancer patients, TLH had better recurrence-free survival than RAH [14]. These findings resemble those of a 2020 Canadian study involving patients with intermediate-risk stage I endometrial cancer, where overall survival between TAH and RAH was similar but recurrence was significantly higher in the RAH group [15].

Due to conflicting evidence in the literature, our study aims to better understand how surgical approaches (laparotomy and laparoscopy) at Kingston Health Sciences Centre (KHSC) impact oncologic and surgical outcomes. Additionally, we assessed various secondary outcomes, including the impact of various molecular profiles on recurrence and overall survival.

## 2. Materials and Methods

### 2.1. Study Design

This study used a retrospective cohort design to evaluate patients who underwent surgical management for endometrial cancer at KHSC in Kingston, Ontario, Canada. Eligible patients were identified between January 2017 and November 2022 using Canadian Classification of Health Interventions (CCI) codes for total hysterectomy procedures. Medical records were reviewed to identify patients with endometrial cancer confirmed on final pathology.

### 2.2. Participants

All patients with histology-confirmed endometrial cancer who underwent hysterectomy during the study period were eligible for inclusion. Standardized chart abstraction was conducted to collect information regarding patient demographics, medical comorbidities, preoperative and postoperative pathology findings, cancer stage, molecular and genetic markers, surgical approach, use and perioperative complications, length of hospital stay and 30-day readmission.

Preoperative physical status was classified according to the American Society of Anesthesiologists (ASA) Physical Status Classification System, as assigned by the treating anesthesiologist on the day of surgery. Surgical complications were graded using the Clavien–Dindo Classification System, with each complication assessed independently as individual patients could experience more than one postoperative complication.

Participants were followed longitudinally through oncology records at KHSC. Follow-up information included recurrence status, survival outcomes, and date of last clinical contact. Patients were considered lost to follow-up if there was no documented oncology follow-up within 5 years following surgery or if follow-up occurred at regional centres where chart access via Connecting Ontario was restricted due to Research Ethics.

### 2.3. Protocols

Patients were grouped according to the route of surgical management. Laparoscopic and robotic-assisted procedures were categorized together as the minimally invasive surgery group and compared with the open abdominal surgery group. Additional subgroup analyses were performed according to final International Federation of Gynecology and Obstetrics (FIGO) stage (Stage I, Stage II, and Stage III/IV) and molecular or genetic classification. The FIGO 2009 [16] staging classification was used at the time of this study. Additionally, pathology review was centralized within our institution at KHSC.

Recurrence-free survival and overall survival were assessed longitudinally. To be included in survival analyses, patients were required to survive at least 6 weeks following their initial surgical intervention. Furthermore, recurrent disease was only classified when identified more than 6 weeks after surgery to distinguish true recurrence from residual disease present at the time of initial treatment. In the recurrence-free survival analysis, the primary outcome was recurrence, with the event date defined as the date recurrence was diagnosed. In the overall survival analysis, death was the outcome of interest, and the event date corresponded to the recorded date of death. Patients without an event were censored at the date of last known follow-up.

Molecular classification was performed using immunohistochemical staining of tumour specimens. Mismatch repair (MMR) status was assessed using MSH2/MSH6 and MLH1/PMS2 expression and categorized as either intact or deficient. Tumours were classified as MMR-deficient if loss of expression was identified in any MMR protein and as MMR-intact if expression of all assessed proteins was retained. Estrogen and progesterone receptor presence was classified as positive and absence as negative. p53 status was similarly evaluated by immunohistochemistry and classified as wild type or aberrant according to established staining patterns. DNA sequencing was used to detect mutations in the POLE gene when indicated. Molecular subgroup analyses were restricted to patients with available molecular testing results; patients with missing molecular data were excluded from analyses. POLE and p53 genes are, more recently, suggested to contribute to endometrial cancer prognosis, so testing for these genes was mostly restricted to high-risk histologies or recently diagnosed cases within our study period.

For survival analyses evaluating MMR status, patients were categorized as high genetic risk (MMR-deficient) or low genetic risk (MMR-intact), while separate models evaluated p53 aberrant versus p53 wild-type tumours.

### 2.4. Statistical Analyses

Patients who did not experience the outcome of interest were censored at the date of their last documented follow-up. Administrative censoring was applied at 5 years following surgery or at the study end date, whichever occurred first. Patients lost to follow-up were censored at the date of their last known clinical encounter.

Categorical variables were summarized as frequencies and percentages, while continuous variables were reported as either mean ± standard deviation or median with interquartile range depending on data distribution.

Comparisons between groups were performed using the chi-square test or Mann–Whitney U test, as appropriate for variable type and distribution. Fisher’s exact test was used when expected cell counts were less than five.

Recurrence-free survival and overall survival were analyzed using Kaplan–Meier survival curves and compared using the log-rank test. Multivariable Cox proportional hazards regression models were subsequently developed to evaluate factors associated with recurrence and mortality, including surgical approach, cancer stage, and molecular or genetic risk classification. Hazard ratios (HRs) with 95% confidence intervals (CIs) were reported, and statistical significance was defined using two-sided *p*-values.

## 3. Results

### 3.1. Patient Characteristics

A total of 341 patients were included in the study, comprising 152 (44.6%) who underwent laparoscopic surgery and 189 (55.4%) who underwent abdominal surgery. The mean age was significantly higher in the laparoscopic group compared with the abdominal group (67.1 ± 10.2 vs. 64.7 ± 11.3 years, *p* = 0.038), while body mass index (BMI) did not differ significantly between groups (34.5 ± 9.2 vs. 34.6 ± 8.7 kg/m^2^, *p* = 0.96). The ASA classification distribution was comparable between groups (*p* = 0.23). In the laparoscopic group, the majority of procedures were laparoscopy (71.1%), with the remainder being vaginal-assisted (14.5%) or robotic-assisted (13.8%) approaches (Table 1).

The rates of additional procedures differed significantly between surgical approaches. Pelvic lymph node dissection, sentinel node mapping, omentectomy and peritoneal washings were more frequent in abdominal cases (*p* = 0.005, <0.001, 0.005, and <0.001, respectively). Paraaortic lymph node dissection was performed in 11.2% of the laparoscopic and 7.4% of the abdominal cases (*p* = 0.26). The intraoperative complication rates were similar between the groups (12.2% vs. 15.5%, *p* = 0.51) (Table 1).

Early postoperative complications occurred more frequently in the abdominal group (37.6%) compared with the laparoscopic group (6.8%) (*p* < 0.001). Most early complications were grade 1 or grade 2 in severity. The median hospital stay was significantly shorter in the laparoscopic group (1 day [IQR 0.5–1]) than in the abdominal group (3 days [IQR 3–5], *p* < 0.001). Readmissions within 30 days were more common following abdominal surgery (5.9% vs. 1.4%, *p* = 0.044). The proportion of patients who died during follow-up was similar between groups (12.5% vs. 12.2%), although the proportion of patients lost to follow-up was higher in the abdominal group (39.9% vs. 19.1%, *p* < 0.001) (Table 1).

### 3.2. Histopathology and Tumour Characteristics

Preoperative endometrial biopsy mostly identified grade 1 or grade 2 endometrioid adenocarcinoma. The laparoscopic group had a higher proportion of grade 1 tumours (28.9% vs. 17.5%, *p* = 0.012), whereas serous carcinoma was more common in the abdominal group (15.3% vs. 7.9%, *p* = 0.036). Final histopathology demonstrated a higher prevalence of low-grade endometrioid tumours among laparoscopic cases (36.8% vs. 18.0%, *p* < 0.001), while serous and clear cell subtypes were more frequent in abdominal cases. Staging differed significantly by surgical approach: 84.0% of laparoscopic patients presented with stage I disease compared to 63.2% of abdominal cases, whereas stages III–IV disease occurred in 7.3% and 28.1%, respectively (*p* < 0.001) (Table 2).

Adjuvant treatment was administered to 46.1% of laparoscopic and 45.7% of abdominal cases. However, missing data was more frequent in the abdominal group due to incomplete documentation. Cases with missing information were therefore not included within the statistical analysis. The use of chemotherapy trended higher in the abdominal group (21.7% vs. 13.8%, *p* = 0.061), while external beam radiotherapy and vaginal brachytherapy were similar between groups (*p* = 0.55 and *p* = 0.33, respectively). No immunotherapy use was reported in the laparoscopic group (Table 2).

### 3.3. Unadjusted Recurrence and Survival Outcomes

The full cohort was under observation for a median of 128.7 [15.4–195.9] weeks, with a significantly shorter median time under observation among the abdominal case group compared to the laparoscopic (*p* = 0.049). Recurrence occurred in 14.8% of laparoscopic and 19.3% of abdominal cases. Median time to recurrence did not differ significantly (67.7 vs. 51.9 weeks, *p* = 0.51). The patterns of recurrence were heterogeneous across both groups, involving vaginal, pelvic, nodal, abdominal, and pulmonary sites (Table 3). In unadjusted analyses there was no significant difference in time to recurrence between surgical approaches (log-rank *p* = 0.39). However, patients with stages III–IV disease demonstrated markedly reduced recurrence-free survival compared to stages I–II (log-rank *p* < 0.001). Similarly, in unadjusted analyses, overall survival did not differ between surgical routes (log-rank *p* = 0.85). However, significantly reduced survival for stages III–IV disease compared to stages I–II (log-rank *p* < 0.001) was observed (Table 3). Therefore, despite more advanced-stage cases comprising the abdominal group, the recurrence rate remained similar across both surgical techniques.

### 3.4. Multivariate Adjusted Recurrence and Survival Results

**Figure 1:**
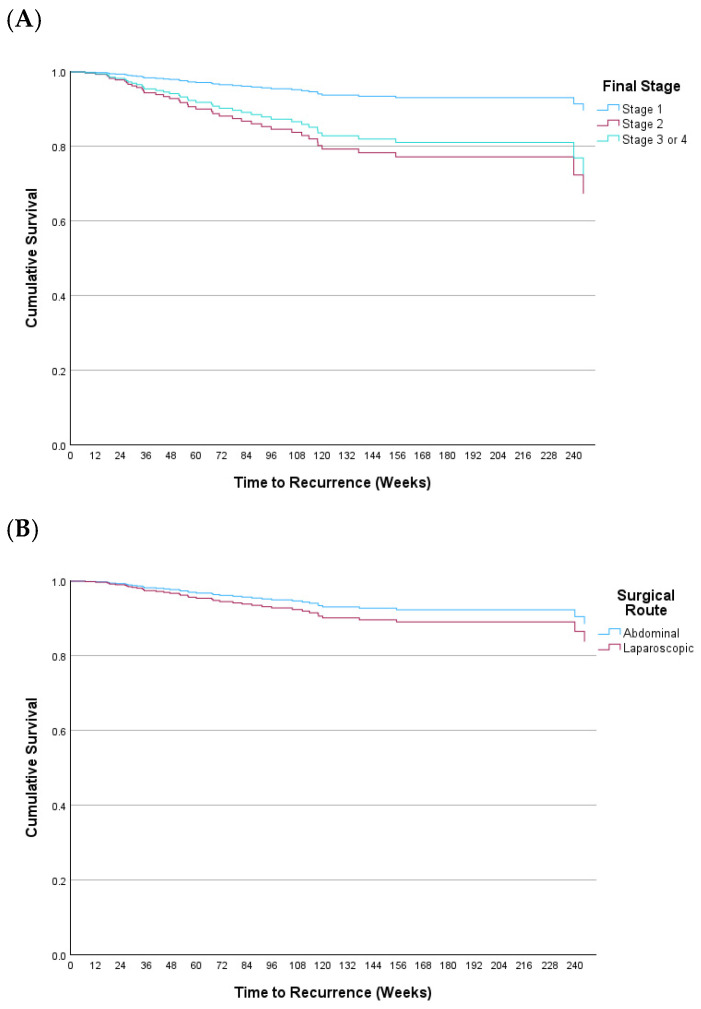
Cox proportional hazards cumulative survival curves for time to recurrence by (**A**) final stage and (**B**) surgical route. Survival tables and number at risk at selected time points are available in Supplemental Section S5.

Recurrence-free survival and OS were examined using a Cox proportional hazards model. In the recurrence-free survival analysis N = 265/341 participants were included, with 40/265 (15.1%) experiencing a recurrence (Table 4). Among those excluded from the analysis, N = 68 had less than 6 weeks of follow-up time, five cases had missing values and three cases were censored prior to the first event. More advanced final stage was associated with significantly reduced time to recurrence compared to stage 1 disease (Figure 1A). Other histology was also associated with significantly reduced time to recurrence compared to high-grade adenocarcinoma. Consistent with the unadjusted analysis, no significant difference in recurrence-free survival time was observed between surgical routes in this multivariate adjusted analysis (Figure 1B).

**Figure 2:**
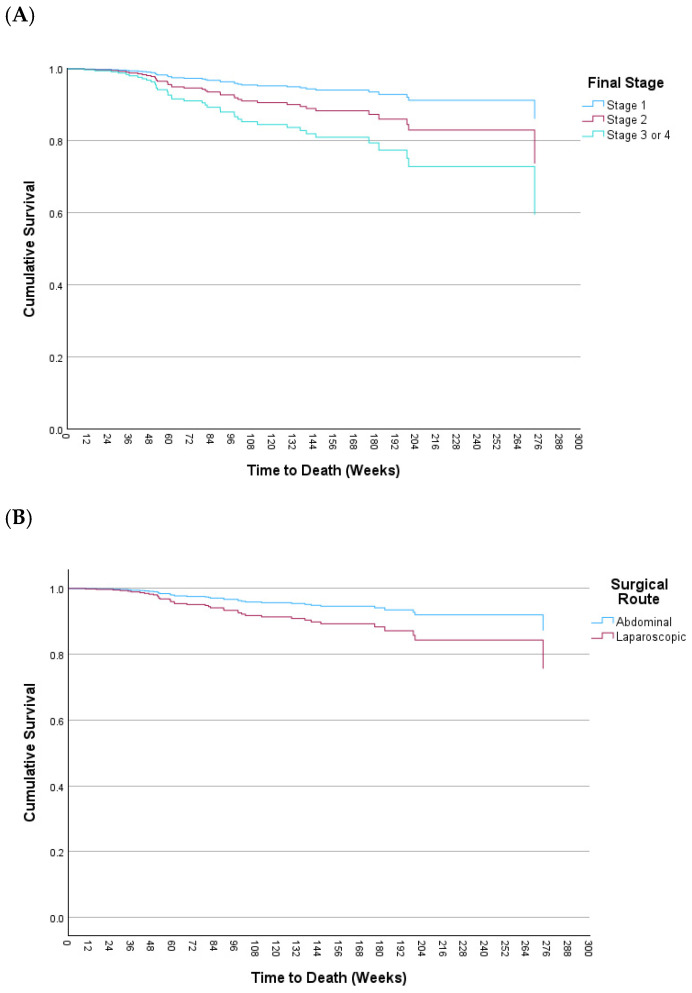
Cox proportional hazards cumulative survival curves for overall survival by (**A**) final stage and (**B**) surgical route. Survival tables and number at risk at selected time points are available in Supplemental Section S6.

In the OS analysis, 257 participants were included, with 40/257 (15.4%) experiencing death during follow-up (Table 5). Among those excluded from the analysis, 68 had less than 6 weeks of follow-up time, five had missing values and 11 were censored prior to the first event. More advanced final stage was associated with significantly reduced time to death compared to stage 1 disease (Figure 2A). Other histology was associated with significantly reduced time to death compared to high-grade adenocarcinoma, while low-grade histology was associated with significantly increased time to death compared to high-grade adenocarcinoma. In the adjusted analysis, the laparoscopic route was associated with significantly reduced time to death compared to the abdominal route (Figure 2B).

### 3.5. Genetic Profiling and Baseline Characteristics

Of the 341 patients with endometrial carcinoma included in this study, 256 (75.1%) underwent comprehensive molecular assessment, while 85 (24.9%) were not genetically evaluated.

Among those tested, MSH2/MSH6 expression was intact in 253 cases (98.8%) and deficient in three (1.2%), whereas MLH/PMS2 was intact in 206 (80.5%) and deficient in 50 (19.5%). Thus, MLH/PMS2 deficiency occurred more frequently than MSH2/MSH6 deficiency. Hormone receptor assessment demonstrated ER positivity in 239 patients (93.4%) and PR positivity in 238 (93.0%). Aberrant P53 expression was detected in 34 cases (13.3%), while 57 (22.3%) showed wild-type staining patterns. Given the novelty of P53 testing during our study period, 165 (64.5%) of the 356 patients with genetic assessment were not assessed for p53. Similarly, POLE mutations were identified in only five cases (2.0%) and not assessed in 223 (87.1%) patients. When stratified by surgical route, the laparoscopic (n = 122) and abdominal (n = 134) groups showed similar molecular distributions, indicating no route-based selection bias for genetic features (Table 6).

### 3.6. Recurrence and Mortality Patterns by Genetic Profile

Among the 256 genetically assessed patients, recurrence patterns varied according to molecular subtype. MSH2/MSH6-deficient tumours demonstrated a recurrence rate of 33.3%, compared with 13.4% among those with intact expression. MLH/PMS2-deficient tumours recurred in 14.0% of cases versus 13.6% of those with intact status. ER-positive and PR-positive tumours demonstrated recurrence rates of 14.2% and 14.3%, respectively. ER-negative and PR-negative tumours demonstrated recurrence rates of 5.9% and 5.6%, respectively. The most striking difference was observed for P53: aberrant P53 expression was associated with a recurrence rate of 38.2%, compared with only 14.0% for wild-type tumours. These findings suggest that P53 aberration, rather than MMR deficiency or hormone receptor profile, is the predominant molecular predictor of recurrence in endometrial carcinoma (Table 7).

The mortality outcomes mirrored the recurrence trends. No deaths occurred among MSH2/MSH6-deficient tumours. MLH/PMS2-deficient tumours exhibited a slightly lower death rate (6.1%) compared to MLH/PMS2-intact cases (15.0%). ER, PR, and POLE statuses were not associated with survival differences. In contrast, P53 aberration was associated with a markedly higher mortality (36.4%) than wild-type expression (7.0%), confirming its strong adverse prognostic impact. Overall, these results underscore P53 as the principal molecular determinant of poor prognosis in this cohort (Table 8).

### 3.7. MMR-Based Genetic Risk and Clinical Outcomes

Patients were further stratified into high-risk (MMR-deficient; n = 53, 20.7%) and low-risk (MMR-intact; n = 203, 79.3%) groups. Recurrence occurred in 15.1% of high-risk versus 13.3% of low-risk cases, while mortality occurred in 5.8% and 15.3%, respectively. These modest and non-significant differences suggest that MMR deficiency, when evaluated independently, does not confer a higher risk of recurrence or death (Table 9).

A multivariable Cox model of time to recurrence was completed among N = 203 patients with known MMR status, with 34/203 (16.7%) experiencing a recurrence during follow-up. MMR deficiency was not associated with a difference in time to recurrence (HR 1.11; 95% CI, 0.41–3.00; *p* = 0.83). In this MMR status-adjusted model, surgical route was not associated with a difference in time to recurrence (HR 1.95; 95% CI, 0.86–4.43; *p* = 0.11), while stages III–IV disease remained associated with significantly reduced time to recurrence (HR 3.86; 95% CI, 1.32–11.29; *p* = 0.014) (Supplemental Section S1).

A corresponding multivariable Cox model of time to death was completed among N = 198 patients with known MMR status, with 33/198 (16.7%) passing away during follow-up. MMR deficiency was not associated with a difference in time to death (HR 1.67; 95% CI, 0.85–3.28; *p* = 0.13). In this MMR status-adjusted model surgical route was not associated with difference in time to death (HR 1.84; 95% CI, 0.83–4.08; *p* = 0.13), while stages III–IV disease remained associated with significantly reduced time to death (HR 3.37; 95% CI, 1.21–9.38; *p* = 0.020) (Supplemental Section S2).

A multivariable Cox model of time to recurrence was completed among N = 68 patients with known P53 status, with 21/68 (30.9%) experiencing a recurrence during follow-up. Aberrant P53 conferred was not associated with a difference in time to recurrence (HR 0.30; 95% CI, 0.06–1.49; *p* = 0.14) (Supplemental Section S3).

A corresponding multivariable Cox model of time to death was completed among N = 66 patients with known P53 status, with 15/66 (22.7%) passing away during follow-up (additional patients were excluded from this analysis due to censoring prior to the earliest event in a stratum). P53 aberration was not associated with a difference in time to death (HR 0.61; 95% CI, 0.09–4.24; *p* = 0.61) (Supplemental Section S4).

### 3.8. Summary of Findings

Overall, molecular profiling revealed that mismatch repair (MMR) deficiency and POLE mutation were not associated with significant differences in recurrence or overall survival. While aberrant p53 expression was associated with substantially higher crude recurrence and mortality rates, these associations were not maintained in adjusted survival analyses, likely reflecting the limited number of patients with available p53 testing and the corresponding reduction in statistical power. Advanced stage at diagnosis remained the most consistent predictor of adverse outcomes across all the models, demonstrating a strong association with both recurrence and mortality.

Notably, laparoscopic surgery was associated with a shorter time to death compared with abdominal surgery after adjustment for clinical and pathological factors. However, surgical route was not associated with recurrence risk and did not demonstrate a significant association with survival in the molecular subgroup analyses. Furthermore, overall survival was high in both surgical groups, suggesting that the absolute difference in outcomes was small despite the observed statistical association. Given the retrospective study design and potential for residual confounding, this finding should be interpreted cautiously and may reflect unmeasured differences in patient, tumour, or treatment characteristics rather than a clinically meaningful effect of surgical approach.

## 4. Discussion

This study evaluated the association between surgical approach, molecular markers, and clinical outcomes in patients with endometrial cancer. Our findings demonstrate that laparoscopic surgery offers comparable oncologic outcomes to abdominal surgery while conferring significant perioperative advantages, including shorter hospital stays and fewer early complications. Among the molecular variables, aberrant p53 expression emerged as the strongest independent predictor of recurrence and mortality. Mismatch repair (MMR) deficiency did not independently predict outcomes, aligning with prior work suggesting that MMR-deficient endometrial carcinoma is not uniformly aggressive [17,18].

Our findings are consistent with extensive research assessing the oncologic safety of minimally invasive surgery (MIS) for endometrial cancer. For instance, the LACE randomized clinical trial demonstrated equivalent disease-free survival (DFS), recurrence rates, and overall survival (OS) between total laparoscopic hysterectomy (TLH) and total abdominal hysterectomy (TAH) over 4.5 years of follow-up in stage I cases [8]. A Cochrane review by Galaal et al. pooling more than 3700 patients also found no significant difference in recurrence between TLH and TAH [19]. Further, Yuan et al. synthesized 37 trials (n = 13,446) in a systematic review and network meta-analysis and found no significant differences in DFS or OS among TAH, TLH, and RAH, although TLH ranked highest for both endpoints [20]. Natarajan et al. concluded that MIS offers similar oncologic outcomes to open surgery while also reducing blood loss, complications, and hospital stay [21].

Comparisons between TLH and RAH remain limited. Chambers et al. published a large retrospective study of 1150 patients, which found no significant differences in progression-free survival (PFS) or OS across multiport, single-port, or robotic-assisted laparoscopy [22]. However, Argenta et al., in a cohort of 1027 stage I patients, reported poorer DFS and OS in the RAH group compared with TLH [23]. Song et al. also reported higher recurrence rates with RAH among intermediate-risk stage I patients receiving adjuvant radiation [15]. These contrasting findings may reflect differences in tumour biology, surgeon experience, institutional volume or unmeasured confounders. Overall, the evidence remains mixed, underscoring the need for prospective multi-institutional studies.

While most of the literature focuses on low-risk endometrial cancer, a database study by Vardar et al. examining high-risk histologies reported no significant differences in long-term oncologic outcomes between TAH and TLH [24]. These findings support the feasibility of MIS even in high-risk cases, although careful patient selection is essential given the potential complexity of staging and cytoreduction.

Recent advances in molecular classification and biomarker-directed therapy have further reshaped the management of high-risk endometrial carcinoma, particularly uterine serous and p53-abnormal subtypes. Uterine serous carcinoma is characterized by aggressive clinical behaviour, frequent p53 alterations and overexpression of HER2/neu in a substantial subset of tumours, creating opportunities for targeted systemic therapy [25]. Recent reviews have highlighted the growing role of HER2-directed treatment strategies, including trastuzumab-based regimens, in improving progression-free and overall survival among patients with HER2-positive serous carcinomas [25]. These developments are clinically relevant to our cohort, where aberrant p53 expression emerged as the strongest molecular predictor of recurrence and mortality in unadjusted models, reinforcing the importance of integrating molecular risk stratification into surgical and adjuvant treatment planning. Our findings also support the evolving paradigm that surgical decision-making in endometrial cancer should increasingly be interpreted within a biomarker-driven framework rather than solely through traditional histopathologic staging. Although minimally invasive surgery demonstrated comparable oncologic outcomes overall, patients with advanced-stage disease and aggressive molecular profiles experienced substantially worse survival outcomes independent of surgical route. This suggests that tumour biology may exert a greater influence on prognosis than operative approach alone. Incorporating molecular markers such as p53 status, MMR deficiency, and HER2 expression into perioperative risk assessment may help to refine individualized treatment strategies, guide adjuvant therapy selection and identify patients who may benefit from targeted or immunotherapeutic approaches in addition to surgical management.

Minimally invasive approaches—including TLH and RAH—consistently demonstrate superior perioperative outcomes compared with laparotomy. Meta-analyses report reduced operative blood loss, fewer complications, and shorter hospitalization with MIS relative to open surgery [14,26]. The results from our study are comparable with those of previously published studies. Comparisons between TLH and RAH remain heterogeneous. While some studies identify comparable perioperative outcomes, others report slightly higher complication rates with RAH—especially in lower-volume centres [27,28]. Despite the mixed findings, both TLH and RAH are widely regarded as safe options, with oncologic risk factors driving surgical decision-making.

Several methodological considerations constrain causal interpretation of the observed associations in our study. First, the retrospective non-randomized design introduces the potential for confounding by indication, whereby surgical route may have been influenced by baseline disease severity, histology, patient comorbidity, surgeon preference or perceived operative complexity. Patients undergoing abdominal hysterectomy demonstrated a greater proportion of advanced-stage and higher-risk histologic disease, which may partially account for differences in recurrence and survival outcomes independent of surgical approach. Although multivariable adjustment was performed, residual confounding from unmeasured clinical and treatment-related variables remains possible. Second, differential follow-up between surgical groups may have introduced attrition bias as loss to follow-up was more frequent among abdominal cases. This may have resulted in under-ascertainment of recurrence or mortality events and could bias survival estimates if the patients lost to follow-up differed systematically from those retained in longitudinal assessment. Third, molecular characterization was incomplete for a substantial proportion of the cohort, particularly for p53, ER, PR, and POLE testing, reflecting evolving institutional testing practices over the study period. Missing molecular data may have reduced statistical power, introduced selection bias, and limited the ability to fully evaluate the independent prognostic contribution of molecular subtypes. Consequently, the findings should be interpreted as associative rather than causal, and prospective studies with standardized molecular testing and longitudinal follow-up are needed to better define the independent effects of surgical approach and molecular risk factors on oncologic outcomes.

Nevertheless, the findings provide valuable real-world evidence on molecularly stratified outcomes. Future research should incorporate molecular profiles such as p53 status, MMR status, and POLE ultramutation, alongside clinical and surgical data, to enhance prognostic models. Prospective comparisons of TLH and RAH with standardized surgical protocols would help to resolve ongoing uncertainty regarding potential differences in recurrence and survival outcomes. Continued adoption of MIS techniques must balance technical feasibility, patient safety, and oncologic outcomes.

## 5. Conclusions

In this study, minimally invasive surgical approaches—particularly total laparoscopic hysterectomy—were associated with favourable perioperative outcomes and oncologic safety comparable to open abdominal surgery across diverse stages and histologic subtypes of endometrial cancer. Laparoscopic surgery demonstrated significantly reduced postoperative morbidity and shorter hospital stays without compromising recurrence-free or overall survival. Molecular profiling further highlighted the dominant prognostic impact of p53 aberration, while mismatch repair deficiency did not independently predict recurrence or survival. These findings reinforce the importance of integrating molecular classification with clinical and surgical factors when counselling patients and making treatment decisions.

Collectively, the results support the continued adoption of minimally invasive hysterectomy as a safe and effective approach in appropriately selected patients, including those with high-risk disease. Future prospective studies with comprehensive molecular characterization are needed to refine individualized risk stratification, clarify differences between laparoscopic and robotic-assisted techniques, and optimize surgical and adjuvant treatment pathways. Such integrated approaches may ultimately enhance personalized care and improve long-term outcomes for patients with endometrial carcinoma.

## Figures and Tables

**Table 1:** Baseline demographic and clinical characteristics of patients undergoing laparoscopic, abdominal, or robotic-assisted hysterectomy for endometrial carcinoma. Data include age, body mass index, comorbidities, tumour stage, and perioperative characteristics. Comparisons were performed across surgical approaches.

	Laparoscopic N = 152	Abdominal N = 189	*p*-Value
Age (years), mean ± SD	67.1 ± 10.2	64.7 ± 11.3	0.038
BMI (kg/m^2^), mean ± SD	34.5 ± 9.2	34.6 ± 8.7	0.96
Missing	2	9	
ASA, n (%)			0.23
1–2	38 (25.2)	39 (20.6)	
3	101 (66.9)	125 (66.1)	
4–5	12 (7.9)	25 (13.2)	
Procedure Type, n (%)			N/A
Total Laparoscopic	108 (71.1)	0	
Vaginal ± Laparoscopic Assist	22 (14.5)	0	
Robot Assist	21 (13.8)	0	
Total Abdominal	0	189 (100.0)	
Additional Procedure Type, n (%)			
BS	1 (0.7)	2 (1.1)	1.00
BSO	143 (94.1)	178 (94.2)	1.00
Omentectomy	25 (16.4)	56 (29.6)	0.005
elvic Lymph Node Dissect (bilateral)	73 (48.0)	100 (52.9)	0.39
Pelvic Lymph Node Dissect (unilateral)	1 (0.7)	11 (5.8)	0.014
Sentinel Node Dissect	21 (13.8)	2 (1.1)	<0.001
Paraaortic Lymph Node Dissect	17 (11.2)	14 (7.4)	0.26
Peritoneal Biopsy	0	1 (0.5)	1.00
Pelvic Washings	8 (5.3)	67 (35.4)	<0.001
Intraoperative Complication, n (%)			0.51
Yes	17 (12.2)	25 (15.5)	
No	122 (87.8)	136 (84.5)	
Missing	13	28	
Early Complication, n (%)			<0.001 *
Yes	10 (6.8)	71 (37.6)	
No	137 (93.2)	118 (62.4)	
Missing	5	0	
Type Early Complication *, n			N/A
Grade 1	8	32	
Grade 2	2	36	
Grade 3a	0	4	
Grade 3b	1	4	
Grade 4a	0	2	
Grade 4b	0	1	
Length of Stay (days), median [IQR]	1 [0.5–1]	3 [3–5]	<0.001
Min–Max	0-49	1–72	
Readmission (within 30 days), n (%)			0.044
Yes	2 (1.4)	11 (5.9)	
No	146 (98.6)	175 (94.1)	
Unknown/NA	4	2	
Death, n (%)			<0.001
Yes	19 (12.5)	23 (12.2)	
No	104 (68.4)	90 (47.9)	
Lost to Follow-Up	29 (19.1)	75 (39.9)	
Missing	0	1	

* Each complication was graded individually; one patient may have had multiple complications.

**Table 2:** Pathologic characteristics and adjuvant treatment patterns stratified by surgical approach. Variables include histologic grade, depth of myometrial invasion, lymphovascular space invasion, nodal assessment, and receipt of adjuvant radiotherapy or chemotherapy.

	Laparoscopic N = 152	Abdominal N = 189	*p*-Value
Preoperative Endobiopsy a, n (%)			
Grade 1 Endometrioid Adenocarcinoma	44 (28.9)	33 (17.5)	0.012
Grade 2 Endometrioid Adenocarcinoma	50 (32.9)	55 (29.1)	0.45
Grade 3 Endometrioid Adenocarcinoma	9 (5.9)	11 (5.8)	0.97
High-Grade Endometrioid Adenocarcinoma	12 (7.9)	14 (7.4)	0.87
Serous Carcinoma	12 (7.9)	29 (15.3)	0.036
Clear Cell Carcinoma	7 (4.6)	9 (4.8)	0.95
Undifferentiated Carcinoma	0	1 (0.5)	1.00
Other	28 (18.4)	47 (24.9)	0.15
Not Done	3	7	
Final Histopathology a, n (%)			
Grade 1 Endometrioid Adenocarcinoma	56 (36.8)	34 (18.0)	<0.001
Grade 2 Endometrioid Adenocarcinoma	52 (34.2)	70 (37.0)	0.59
Grade 3 Endometrioid Adenocarcinoma	10 (6.6)	19 (10.1)	0.25
High-Grade Endometrioid Adenocarcinoma	6 (3.9)	11 (5.8)	0.43
Serous Carcinoma	15 (9.9)	28 (14.8)	0.17
Clear Cell Carcinoma	7 (4.6)	9 (4.8)	0.95
Undifferentiated Carcinoma	1 (0.7)	3 (1.6)	0.43
Other	15 (9.9)	47 (24.9)	<0.001
Final Stage, n (%)			<0.001
Stage 1a	81 (54.0)	78 (42.2)	
Stage 1b	45 (30.0)	39 (21.1)	
Stage 2	13 (8.7)	16 (8.6)	
Stage 3a	5 (3.3)	13 (7.0)	
Stage 3b	0	4 (2.2)	
Stage 3c1	5 (3.3)	19 (10.3)	
Stage 3c2	1 (0.7)	4 (2.2)	
Stage 4a	0	3 (1.6)	
Stage 4b	0	9 (4.9)	
Missing	2	4	
Final Stage, n (%)			<0.001
Stage 1	126 (84.0)	117 (63.2)	
Stage 2	13 (8.7)	16 (8.6)	
Stage 3	11 (7.3)	40 (21.6)	
Stage 4	0	12 (6.5)	
Missing	2	4	
Final Stage, n (%)			<0.001
Stage 1	126 (84.0)	117 (63.2)	
Stage 2	13 (8.7)	16 (8.6)	
Stage 3 or 4	11 (7.3)	52 (28.1)	
Missing	2	4	
Adjuvant Therapy, n (%)			<0.001
Yes	70 (46.1)	86 (45.7)	
No	71 (46.7)	56 (29.8)	
N/A	11 (7.2)	46 (24.5)	
Unknown	0	1	
Type Adjuvant Therapy, n (%)			
Pelvic Radiotherapy, External Beam	43 (28.3)	48 (25.4)	0.55
Brachytherapy, Vaginal	32 (21.1)	32 (16.9)	0.33
Chemotherapy	21 (13.8)	41 (21.7)	0.061
Immunotherapy	0	1 (0.5)	1.00
Other	1 (0.78)	3 (1.6)	0.63

**Table 3:** Recurrence outcomes according to surgical approach. Outcomes include recurrence rates, time to recurrence, and overall survival.

	Laparoscopic N = 152	Abdominal N = 189	*p*-Value
Time Under Observation (weeks), median [IQR]	150.7 [80.6–183.7]	99.4 [5.6–220.4]	0.049
Recurrence, n (%)			0.39
Yes	19 (14.8)	22 (19.3)	
No	109 (85.2)	92 (80.7)	
Lost to Follow-Up	24	75	
	Laparoscopic N = 19	Abdominal N = 22	*p*-Value
Age at Recurrence (years), median [IQR]	73 [66–79]	71 [67–79]	0.76
Time to Recurrence (weeks), median [IQR]	67.7 [34.0–110.1]	51.9 [28.7–88.5]	0.51
Recurrence Site a, n			--
Vagina	3	7	
Pelvis	3	3	
Pelvic Lymph Nodes	2	1	
Paraaortic Lymph Nodes	3	5	
Abdomen	9	5	
Lungs	7	6	
Liver	3	1	
Other	5	6	
Recurrence Therapy b, n			--
Surgery	2	3	
Chemotherapy	10	12	
Radiotherapy	4	6	
Immunotherapy	4	1	
No Therapy	5	3	
Other	1	1	

**Table 4:** Cox proportional hazards model of recurrence-free survival.

	Sample in Model N = 265	Hazard Ratio	95% CI		*p*-Value
			Lower	Upper	
Age (years), mean ± SD	65.7 ± 10.9	1.04	0.99	1.08	0.068
ASA (1–5), median [IQR]	3 [2.5–3]	1.55	0.78	3.06	0.21
Surgical Year (2017–2023), median [IQR]	2018 [2018–2019]	1.02	0.80	1.30	0.88
Percent Myometrial Invasion (%), mean ± SD	42.1 ± 33.2	1.01	0.99	1.03	0.13
LVSI, n (%)					
Yes	70 (26.4)	1.0 (ref)	-	-	-
No	171 (64.5)	0.47	0.20	1.13	0.092
Indeterminate	24 (9.1)	0.91	0.35	2.35	0.84
Nodes Collected, n (%)					
Yes	144 (54.3)	1.0 (ref)	-	-	-
No	121 (45.7)	1.61	0.71	3.67	0.25
Adjuvant Therapy, n (%)					
Yes	150 (56.6)	1.0 (ref)	-	-	-
No	115 (43.4)	1.83	0.83	4.05	0.14
Surgical Route, n (%)					
Abdominal	136 (51.3)	1.0 (ref)	-	-	-
Laparoscopic	129 (48.7)	1.45	0.71	2.95	0.31
Final Stage, n (%)					
1	198 (74.7)	1.0 (ref)	-	-	-
2	21 (7.9)	3.62	1.30	10.06	0.014
3 or 4	46 (7.9)	2.93	1.18	7.28	0.020
Final Histology, n (%)					
High-Grade Adenocarcinoma	114 (43.0)	1.0 (ref)	-	-	-
Low-Grade Adenocarcinoma	81 (30.6)	0.22	0.05	1.04	0.055
Other	70 (26.4)	2.31	1.13	4.72	0.021

**Table 5:** Cox proportional hazards model of overall survival.

	Sample in Model N = 257	Hazard Ratio	95.0% CI		*p*-Value
			Lower	Upper	
Age (years), mean ± SD	66.1 ± 10.6	0.99	0.95	1.03	0.56
ASA (1–5), median [IQR]	3 [2.5–3]	1.62	0.83	3.16	0.21
Surgical Year (2017–2023), median [IQR]	2018 [2018–2019]	0.94	0.73	1.21	0.62
Percent Myometrial Invasion (%), mean ± SD	42.4 ± 33.2	1.02	1.004	1.04	0.016
LVSI, n (%)					
Yes	69 (26.8)	1.0 (ref)	-	-	-
No	164 (63.8)	0.43	0.16	1.16	0.095
Indeterminate	24 (9.3)	1.19	0.45	3.15	0.73
Nodes Collected, n (%)					
Yes	138 (53.7)	1.0 (ref)	-	-	-
No	119 (46.3)	4.64	2.09	10.30	<0.001
Adjuvant Therapy, n (%)					
Yes	150 (58.4)	1.0 (ref)	-	-	-
No	107 (41.6)	3.65	1.76	7.57	<0.001
Surgical Route, n (%)					
Abdominal	128 (49.8)	1.0 (ref)	-	-	-
Laparoscopic	129 (50.2)	2.05	1.02	4.11	0.044
Final Stage, n (%)					
1	192 (74.7)	1.0 (ref)	-	-	-
2	21 (8.2)	2.04	0.68	6.10	0.20
3 or 4	44 (17.1)	3.47	1.34	9.00	0.010
Final Histology, n (%)					
High-Grade Adenocarcinoma	108 (42.0)	1.0 (ref)	-	-	-
Low-Grade Adenocarcinoma	79 (30.7)	0.25	0.075	0.84	0.025
Other	70 (27.2)	2.35	1.05	5.28	0.038

**Table 6:** Distribution of molecular features by surgical route. Molecular alterations assessed include mismatch repair (MMR) status and p53 expression, demonstrating no significant differences across surgical approaches.

	Genetics Assessed N = 256	Laparoscopic N = 122	Abdominal N = 134
MSH2/MSH6			
Intact	253 (98.8)	122 (100.0)	131 (97.8)
Deficient	3 (1.2)	0 (0.0)	3 (2.2)
MLH/PMS2			
Intact	206 (80.5)	95 (77.9)	111 (82.8)
Deficient	50 (19.5)	27 (22.1)	23 (17.2)
ER			
Positive	239 (93.4)	117 (95.9)	122 (91.0)
Negative	17 (6.6)	5 (4.1)	12 (9.0)
PR			
Positive	238 (93.0)	116 (95.1)	122 (91.0)
Negative	18 (7.0)	6 (4.9)	12 (9.0)
P53			
Wild Type	57 (22.3)	311 (25.4)	26 (19.4)
Aberrant	34 (13.3)	11 (9.0)	23 (17.2)
Unknown	165 (64.5)	80 (65.6)	85 (63.4)
POLE			
Mutated	5 (2.0)	3 (2.5)	2 (1.5)
Not Mutated	28 (10.9)	20 (16.4)	8 (6.0)
Unknown	223 (87.1)	99 (81.1)	124 (92.5)

**Table 7:** Recurrence outcomes stratified by molecular classification. Associations between molecular subtype and recurrence risk are shown, including univariable and multivariable analyses.

	Genetics Assessed N = 256 (Column %)	Recurrence n/N (%)
MSH2/MSH6		
Intact	253 (98.8)	34/253 (13.4)
Deficient	3 (1.2)	1/3 (33.3)
MLH/PMS2		
Intact	206 (80.5)	28/206 (13.6)
Deficient	50 (19.5)	7/50 (14.0)
ER		
Positive	239 (93.4)	34/239 (14.2)
Negative	17 (6.6)	1/17 (5.9)
PR		
Positive	238 (93.0)	34/238 (14.3)
Negative	18 (7.0)	1/18 (5.6)
P53		
Wild Type	57 (22.3)	8/57 (14.0)
Aberrant	34 (13.3)	13/34 (38.2)
Unknown	165 (64.5)	14/165 (8.5)
POLE		
Mutated	5 (2.0)	1/5 (20.0)
Not Mutated	28 (10.9)	4/28 (14.3)
Unknown	223 (87.1)	30/223 (13.5)

**Table 8:** Overall survival outcomes by molecular classification. Mortality events and survival estimates are presented according to MMR and p53 status.

	Genetics Assessed N = 256 (Column %)	Death n/N (%)
MSH2/MSH6		
Intact	253 (98.8)	34/253 (13.5)
Deficient	3 (1.2)	0/3 (0.0)
MLH/PMS2		
Intact	206 (80.5)	31/206 (15.0)
Deficient	50 (19.5)	3/50 (6.1)
ER		
Positive	239 (93.4)	32/239 (13.4)
Negative	17 (6.6)	2/17 (11.8)
PR		
Positive	238 (93.0)	31/238 (13.1)
Negative	18 (7.0)	3/18 (16.7)
P53		
Wild Type	57 (22.3)	4/57 (7.0)
Aberrant	34 (13.3)	12/34 (36.4)
Unknown	165 (64.5)	18/165 (10.9)
POLE		
Mutated	5 (2.0)	0/5 (0.0)
Not Mutated	28 (10.9)	2/28 (7.1)
Unknown	223 (87.1)	32/223 (14.4)

**Table 9:** Recurrence and survival outcomes among high-risk molecular subgroups. High-risk groups include MMR-deficient and p53-aberrant tumours, with outcomes stratified accordingly.

	N = 256 (Column %)	Recurrence n/N (%)	Death n/N (%)
Genetic Risk			
MSH2/MSH6 or MLH/PMS2-deficient	53 (20.7)	8/53 (15.1)	3/53 (5.8)
MSH2/MSH6 and MLH/PMS2-intact	203 (79.3)	27/203 (13.3)	31/203 (15.3)

## Data Availability

The raw data supporting the conclusions of this article will be made available by the authors on request.

## Supplementary Materials

The following supporting information can be downloaded at: https://www.mdpi.com/article/10.3390/cancers18121977/s1.

## Abbreviations

The following abbreviations are used in this manuscript:

DOAJ	Directory of Open-Access Journals
MMR	Mismatch Repair
TLH	Total Laparoscopic Hysterectomy
RAH	Robotic-Assisted Hysterectomy
TAH	Total Abdominal Hysterectomy
MIS	Minimally Invasive Surgery
