# Supplementary material for: Retrospective Cohort Study Comparing Different Hysterectomy Approaches for the Treatment of Endometrial Cancer
Source: Cancers (Basel). 2026 Jun 18;18(12):1977. doi: 10.3390/cancers18121977 (PMC13296435; doi:10.3390/cancers18121977)
Supplement: Supplementary file 1 [file cancers-18-01977-s001.zip › cancers-4118652-supplementary.pdf]

Supplemental S1. Cox Proportional Hazards Model of time to recurrence for patients with known MMR status (N=203).

	Hazard Ratio	95.0% CI for Exp(B)		P-Value
		Lower	Upper	
**MMR Status**				
MMR Intact	1.0 (ref)	-	-	-
MMR Deficient	0.95	0.58	1.56	0.83
**Age** (years)	1.05	1.001	1.10	**0.047**
**ASA** (1-5)	1.68	0.77	3.67	0.19
**Surgical Year** (2017-2023)	0.97	0.76	1.26	0.84
**Percent Myometrial Invasion** (%)	1.02	1.002	1.04	**0.030**
**LVSI**				
Yes	1.0 (ref)	-	-	-
No	0.82	0.27	2.55	0.74
Indeterminate	0.81	0.26	2.55	0.72
**Nodes Collected**				
Yes	1.0 (ref)	-	-	-
No	1.27	0.49	3.29	0.62
**Adjuvant Therapy**				
Yes	1.0 (ref)	-	-	-
No	1.81	0.66	4.95	0.25
**Surgical Route**				
Abdominal	1.0 (ref)	-	-	-
Laparoscopic	1.95	0.86	4.43	0.11
**Final Stage**				
1	1.0 (ref)	-	-	-
2	3.45	0.85	13.91	0.082
3 or 4	3.86	1.32	11.29	**0.014**
**Final Histology**				
High Grade Adenocarcinoma	1.0 (ref)	-	-	-
Low Grade Adenocarcinoma	0.21	0.043	1.03	0.054
Other	2.38	0.95	5.96	0.064

Supplemental S2. Cox Proportional Hazards Model of overall survival for patients with known MMR status (N=198).

	Hazard Ratio	95.0% CI for Exp(B)		P-Value
		Lower	Upper	
**MMR Status**				
MMR Intact	1.0 (ref)	-	-	-
MMR Deficient	1.67	0.85	3.28	0.13
**Age** (years)	0.99	0.94	1.03	0.54
**ASA** (1-5)	2.18	1.05	4.53	**0.038**
**Surgical Year** (2017-2023)	0.96	0.64	1.15	0.32
**Percent Myometrial Invasion** (%)	1.02	1.001	1.03	**0.041**
**LVSI**				
Yes	1.0 (ref)	-	-	-
No	0.40	0.13	1.19	0.099
Indeterminate	1.19	0.39	3.65	0.76
**Nodes Collected**				
Yes	1.0 (ref)	-	-	-
No	4.78	1.90	12.00	**<0.001**
**Adjuvant Therapy**				
Yes	1.0 (ref)	-	-	-
No	2.99	1.33	6.72	**0.008**
**Surgical Route**				
Abdominal	1.0 (ref)	-	-	-
Laparoscopic	1.84	0.83	4.08	0.13
**Final Stage**				
1	1.0 (ref)	-	-	-
2	1.47	0.38	5.68	0.58
3 or 4	3.37	1.21	9.38	**0.020**
**Final Histology**				
High Grade Adenocarcinoma	1.0 (ref)	-	-	-
Low Grade Adenocarcinoma	0.21	0.059	0.74	**0.015**
Other	2.03	0.75	5.47	0.16

Supplemental S3. Cox Proportional Hazards Model of time to recurrence for patients with known P53 status (N=68).

	**Hazard Ratio**	**95.0% CI for Exp(B)**		**P-Value**
		**Lower**	**Upper**	
**P53 Status**				
Wild type	1.0 (ref)	-	-	-
Aberrant	0.30	0.060	1.49	0.14
**Age** (years)	1.14	1.04	1.24	**0.003**
**ASA** (1-5)	0.83	0.24	2.89	0.77
**Surgical Year** (2017-2023)	0.79	0.58	1.07	0.13
**Percent Myometrial Invasion** (%)	0.99	0.96	1.01	0.30
**LVSI**				
Yes	1.0 (ref)	-	-	-
No	0.58	0.13	2.59	0.48
Indeterminate	2.45	0.50	11.95	0.27
**Nodes Collected**				
Yes	1.0 (ref)	-	-	-
No	12.03	2.27	63.77	**0.003**
**Adjuvant Therapy**				
Yes	1.0 (ref)	-	-	-
No	0.46	0.093	2.28	0.34
**Surgical Route**				
Abdominal	1.0 (ref)	-	-	-
Laparoscopic	1.31	0.39	4.38	0.67
**Final Stage**				
1	1.0 (ref)	-	-	-
2	96.09	8.36	1104.24	**<0.001**
3 or 4	32.50	3.94	268..41	**0.001**
**Final Histology**				
High Grade Adenocarcinoma	1.0 (ref)	-	-	-
Low Grade Adenocarcinoma	0.50	0.079	3.21	0.47
Other	1.47	0.35	6.23	0.60

Supplemental S4. Cox Proportional Hazards Model of overall survival for patients with known P53 status (N=66).

	Hazard Ratio	95.0% CI for Exp(B)		P-Value
		Lower	Upper	
**P53 Status**				
Wild type	1.0 (ref)	-	-	-
Aberrant	0.61	0.087	4.24	0.61
**Age** (years)	1.01	0.92	1.11	0.80
**ASA** (1-5)	1.19	0.23	6.07	0.84
**Surgical Year** (2017-2023)	0.80	0.55	1.18	0.26
**Percent Myometrial Invasion** (%)	1.02	0.98	1.03	0.28
**LVSI**				
Yes	1.0 (ref)	-	-	-
No	0.57	0.08	4.11	0.58
Indeterminate	0.81	0.073	9.01	0.86
**Nodes Collected**				
Yes	1.0 (ref)	-	-	-
No	7.59	1.02	56.70	**0.048**
**Adjuvant Therapy**				
Yes	1.0 (ref)	-	-	-
No	0.78	0.13	4.70	0.78
**Surgical Route**				
Abdominal	1.0 (ref)	-	-	-
Laparoscopic	1.61	0.29	8.80	0.58
**Final Stage**				
1	1.0 (ref)	-	-	-
2	6.01	0.50	72.84	0.16
3 or 4	10.17	1.41	73.40	**0.021**
**Final Histology**				
High Grade Adenocarcinoma	1.0 (ref)	-	-	-
Low Grade Adenocarcinoma	1.00	0.098	10.2	0.99
Other	2.37	0.26	21.56	0.45

Supplemental S5. Time to recurrence by (A) final stage and (B) surgical route.

A1. # of Cumulative Events / # at Risk at selected time points

Time (weeks) →<br>Final Stage↓	6	12	24	36	48	60	72	84	96	108
1	0/198	1/191	3/184	4/179	6/174	7/168	7/167	8/162	10/154	10/147
2	0/21	0/20	1/19	4/16	5/15	5/14	5/14	6/13	7/12	7/12
3 or 4	0/46	1/41	2/37	6/30	6/28	10/24	13/21	13/21	13/21	14/17

Final Stage	120	132	144	156	168	180	192	204	216	228	240
1	13/137	13/127	13/121	14/109	14/91	14/81	14/71	14/63	14/56	14/44	15/27
2	7/12	7/12	7/12	7/12	7/11	7/8	7/7	7/7	7/7	7/5	7/5
3 or 4	16/14	16/13	17/12	17/12	17/8	17/7	17/7	17/7	17/5	17/4	17/4

A2. Survival Table of recurrence events.

Final Stage	Time	Cumulative Proportion Surviving at the Time		N of Cumulative Events	N of Remaining at Risk
		Estimate	Std. Error		
Stage 1	11.857	0.995	0.005	1	191
	17.286	0.990	0.007	2	189
	21.286	0.984	0.009	3	187
	34.000	0.979	0.010	4	180
	40.571	0.973	0.012	5	176
	47.143	0.968	0.013	6	174
	59.714	0.962	0.014	7	168
	81.429	0.956	0.015	8	163
	91.000	0.950	0.016	9	157
	95.571	0.944	0.017	10	154
	110.143	0.938	0.018	11	145
	113.571	0.931	0.019	12	139
	119.857	0.924	0.020	13	137
	155.000	0.916	0.022	14	111
	239.857	0.884	0.037	15	28
Stage 2	18.143	0.950	0.049	1	19
	26.571	0.900	0.067	2	18
	31.429	0.850	0.080	3	17
	34.571	0.800	0.089	4	16
	44.286	0.750	0.097	5	15
	77.143	0.696	0.104	6	13
	86.143	0.643	0.109	7	12
Stage 3 or 4	6.857	0.978	0.022	1	45
	18.429	0.954	0.032	2	39
	25.857	0.928	0.040	3	36
	27.286	0.902	0.047	4	35

	29.143	0.876	0.052	5	33
	35.000	0.847	0.058	6	30
	51.571	0.817	0.063	7	27
	52.143	0.787	0.068	8	26
	55.714	0.757	0.071	9	25
	56.000	0.726	0.075	10	24
	67.143	0.696	0.078	11	23
	67.714	0.666	0.080	12	22
	70.857	0.636	0.082	13	21
	105.429	0.602	0.084	14	18
	117.714	0.565	0.087	15	15
	117.857	0.527	0.089	16	14
	137.286	0.486	0.091	17	12
	244.429	0.365	0.125	18	3

B1. # of Cumulative Events / # at Risk at selected time points

Time (weeks) → / Route↓	6	12	24	36	48	60	72	84	96	108
Abdominal	0/127	0/126	3/117	8/108	9/102	13/95	14/95	15/91	17/88	17/82
Laparoscopic	0/129	2/126	3/123	6/117	8/115	10/109	11/107	12/105	13/99	14/95

120	132	144	156	168	180	192	204	216	228	240
19/78	19/74	20/71	20/68	20/62	20/59	20/56	20/52	20/47	20/37	20/27
17/85	17/78	17/75	18/65	18/48	18/37	18/29	18/25	18/21	18/16	19/9

B2. Survival Table of recurrence events.

Route	Time	Cumulative Proportion Surviving at the Time		N of Cumulative Events	N of Remaining at Risk
		Estimate	Std. Error		
Abdominal	17.286	0.992	0.008	1	123
	18.143	0.984	0.011	2	122
	18.429	0.976	0.014	3	121
	25.857	0.967	0.016	4	116
	27.286	0.959	0.018	5	115
	29.143	0.951	0.020	6	113
	31.429	0.942	0.021	7	110
	34.571	0.934	0.023	8	108
	47.143	0.925	0.024	9	103
	51.571	0.915	0.026	10	100
	52.143	0.906	0.027	11	99

	55.714	0.897	0.028	12	97
	56.000	0.888	0.029	13	96
	67.143	0.878	0.031	14	94
	81.429	0.869	0.032	15	92
	86.143	0.859	0.033	16	90
	95.571	0.850	0.034	17	88
	117.714	0.839	0.035	18	79
	117.857	0.828	0.036	19	78
	137.286	0.817	0.037	20	72
	244.429	0.784	0.048	21	24
Laparoscopic	6.857	0.992	0.008	1	128
	11.857	0.984	0.011	2	126
	21.286	0.977	0.013	3	125
	26.571	0.969	0.015	4	122
	34.000	0.961	0.017	5	119
	35.000	0.953	0.019	6	118
	40.571	0.944	0.020	7	116
	44.286	0.936	0.022	8	115
	59.714	0.928	0.023	9	111
	67.714	0.919	0.024	10	109
	70.857	0.911	0.026	11	108
	77.143	0.903	0.027	12	106
	91.000	0.894	0.028	13	101
	105.429	0.884	0.029	14	96
	110.143	0.875	0.030	15	94
	113.571	0.865	0.032	16	87
	119.857	0.855	0.033	17	85
	155.000	0.842	0.035	18	66
	239.857	0.766	0.080	19	10

Supplemental S6. Overall survival by (A) final stage and (B) surgical route.

A1. # of Cumulative Events / # at Risk at selected time points

Time (weeks) → / Final Stage↓	6	12	24	36	48	60	72	84	96	108	120	132
1	0/191	0/190	0/186	1/182	6/177	7/173	9/169	9/166	11/160	12/152	12/144	12/134
2	0/21	1/20	1/20	1/20	2/19	3/18	3/17	3/17	3/17	3/17	4/16	5/15
3 or 4	0/44	1/42	3/39	6/34	8/32	9/31	9/31	12/26	15/23	16/19	16/18	16/16

Final Stage	144	156	168	180	192	204	216	228	240	252	264	276	288	300
1	12/129	13/116	13/97	13/85	14/75	15/64	15/57	15/45	15/28	15/24	16/13	16/3	16/0	16/0
2	5/15	5/14	5/13	6/9	6/8	6/8	6/8	6/6	6/6	6/4	6/4	6/3	6/1	6/1
3 or 4	17/14	17/13	17/8	17/7	17/7	17/7	17/5	17/4	17/4	17/4	17/3	17/3	17/1	17/0

A2. Survival Table of recurrence events.

Final Stage	Time	Cumulative Proportion Surviving at the Time		N of Cumulative Events	N of Remaining at Risk
		Estimate	Std. Error		
Stage 1	35.714	0.995	0.005	1	182
	37.571	-	-	2	181
	37.571	0.984	0.009	3	180
	40.143	0.978	0.011	4	179
	41.143	0.973	0.012	5	178
	48.857	0.967	0.013	6	177
	58.143	0.962	0.014	7	173
	63.714	0.956	0.015	8	172
	66.857	0.951	0.016	9	170
	88.143	0.945	0.017	10	165
	89.429	0.939	0.018	11	164
	97.571	0.933	0.019	12	159
	145.571	0.926	0.020	13	128
	182.143	0.915	0.022	14	84
	195.143	0.902	0.025	15	71
	263.143	0.846	0.060	16	15
Stage 2	8.000	0.952	0.046	1	20
	46.143	0.905	0.064	2	19
	58.286	0.857	0.076	3	18
	114.714	0.807	0.087	4	16
	128.429	0.756	0.095	5	15
	176.286	0.693	0.106	6	11
Stage 3 or 4	9.714	0.977	0.022	1	43
	14.857	0.954	0.032	2	41
	24.286	0.930	0.039	3	39
	27.286	0.906	0.045	4	38

	30.286	0.882	0.050	5	36
	31.714	0.857	0.054	6	35
	41.143	0.832	0.058	7	33
	48.000	0.807	0.061	8	32
	58.143	0.782	0.065	9	31
	78.714	0.756	0.067	10	29
	81.000	0.730	0.070	11	28
	81.857	0.703	0.072	12	26
	90.143	0.676	0.074	13	25
	91.286	0.648	0.076	14	24
	96.714	0.621	0.078	15	23
	101.857	0.592	0.079	16	20
	139.714	0.552	0.083	17	14

B1. # of Cumulative Events / # at Risk at selected time points

Time (weeks) → / Route↓	6	12	24	36	48	60	72	84	96	108	120	132
Abdominal	0/127	1/125	3/119	6/113	12/107	13/104	13/103	14/98	17/95	18/87	18/86	18/82
Laparoscopic	0/129	1/128	1/126	2/123	3/122	6/118	8/114	10/111	12/105	13/101	14/92	15/83

144	156	168	180	192	204	216	228	240	252	264	276	288	300
19/78	19/74	19/66	20/61	20/58	21/53	21/48	21/38	21/28	21/24	21/15	21/8	21/4	21/1
15/80	16/69	16/52	16/40	17/32	17/26	17/22	17/17	17/10	17/8	18/5	18/1	18/0	18/0

B2. Survival Table of recurrence events.

Route	Time	Cumulative Proportion Surviving at the Time		N of Cumulative Events	N of Remaining at Risk
		Estimate	Std. Error		
Abdominal	9.714	0.992	0.008	1	126
	14.857	0.984	0.011	2	124
	24.286	0.976	0.014	3	119
	27.286	0.968	0.016	4	118
	30.286	0.959	0.018	5	115
	31.714	0.951	0.019	6	114
	37.571	0.943	0.021	7	112
	40.143	0.934	0.022	8	111
	41.143	-	-	9	110
	41.143	0.917	0.025	10	109
	46.143	0.909	0.026	11	108
	48.000	0.901	0.027	12	107
	48.857	0.892	0.028	13	106

	81.000	0.883	0.029	14	100
	89.429	0.874	0.030	15	97
	90.143	0.865	0.031	16	96
	91.286	0.856	0.032	17	95
	101.857	0.847	0.033	18	91
	139.714	0.836	0.035	19	79
	176.286	0.823	0.036	20	63
	195.143	0.809	0.038	21	56
Laparoscopic	8.000	0.992	0.008	1	128
	35.714	0.984	0.011	2	123
	37.571	0.976	0.014	3	122
	58.143	-	-	4	120
	58.143	0.960	0.017	5	119
	58.286	0.952	0.019	6	118
	63.714	0.944	0.021	7	117
	66.857	0.936	0.022	8	115
	78.714	0.928	0.023	9	113
	81.857	0.919	0.024	10	111
	88.143	0.911	0.026	11	110
	96.714	0.902	0.027	12	105
	97.571	0.894	0.028	13	104
	114.714	0.884	0.029	14	93
	128.429	0.874	0.030	15	87
	145.571	0.863	0.032	16	78
	182.143	0.842	0.038	17	39
	263.143	0.721	0.116	18	6
